# Anti-Inflammatory Properties of Irisin, Mediator of Physical Activity, Are Connected with TLR4/MyD88 Signaling Pathway Activation

**DOI:** 10.3390/ijms18040701

**Published:** 2017-03-25

**Authors:** Agnieszka Irena Mazur-Bialy, Ewa Pocheć, Marcin Zarawski

**Affiliations:** 1 Department of Ergonomics and Exercise Physiology, Faculty of Health Science, Jagiellonian University Medical College, Grzegorzecka 20, 31-531 Krakow, Poland; 2 Department of Glycoconjugate Biochemistry, Institute of Zoology, Jagiellonian University, Gronostajowa 9, 30-387 Krakow, Poland; ewa.pochec@uj.edu.pl; 3 Department of Gynecology and Obstetrics with Gynecologic Oncology, Rydygier Hospital Krakow, Zlotej Jesieni 1, 31-826 Krakow, Poland; mzarawski@gmail.com

**Keywords:** physical exercise, irisin, adipomiokine, macrophages, inflammation, sport, leukocytes

## Abstract

Irisin, an adipomiokine known as a mediator of physical activity, induces the browning of adipose tissue and it has potentially protective properties in the development of obesity-related states, such as insulin resistance, arteriosclerosis, and type 2 diabetes. Despite numerous studies conducted on this factor, still little is known about its impact on the functioning of immunocompetent cells, but its potential anti-inflammatory properties were previously suggested. In the current study we investigated the role of irisin (0–100 nM) in the downstream pathway activation of Toll-like receptor 4 (TLR4) in RAW 264.7 macrophages stimulated with lipopolysaccharide (LPS; 100 ng/mL). The results have shown that irisin in high concentrations (50, 100 nM) significantly decreased the TLR4 and MyD88 protein levels, as well as the phosphorylation of nuclear factor-κB (NF-κB), consequently leading to the reduction in the release of crucial pro-inflammatory cytokines. The above was confirmed for interleukin 1β (IL-1β), tumor necrosis factor α (TNFα), interleukin 6 (IL-6), keratinocyte chemoattractant (KC), monocyte chemotactic protein 1 (MCP-1), as well as for high mobility group box 1 (HMGB1). Moreover, our results indicate that this effect is connected with irisin’s impact on the phosphorylation of mitogen-activated protein kinases (MAPKs), where a significant reduction in p-JNK and p-ERK but not p-p38 was observed. In conclusion, these data suggest that irisin has potentially anti-inflammatory properties connected with the downregulation of downstream pathways of TLR4/MyD88.

## 1. Introduction

Exercise-driven activity of skeletal muscles leads to a release of low-molecular-mass proteins called myokines [1]. Irisin is a part of the muscle secretome and it is formed by the proteolytic cleavage of the membrane-bound fibronectin type III domain containing five proteins (FNDC5) [2,3,4]. Irisin plays a pleiotropic role in the metabolism [5]. It acts through autocrine and endocrine signaling [6]. The main endocrine targets for irisin are adipocytes residing in white adipose tissue (WAT) [7]. It has been well documented that irisin regulates adipose tissue–mediated thermogenesis [8] and induces the browning of WAT [9,10,11], resulting in loss of body weight [10]. Irisin was also found to be secreted by adipocytes in a rat [12,13] or mouse model, as well as by human adipose tissue [14].

A sedentary lifestyle accompanied by an energy over-consumption is one of the major reasons for overweight and obesity. This state is associated with the activation of pro-inflammatory pathways and consequently can lead to the development of insulin resistance or other metabolic diseases [15]. Moreover, excessive fat accumulation in adipose tissue is defined as the main reason for the development of obesity-related mild inflammation which, in turn, plays a key role in the development of obesity-associated diseases. The adipose tissue is infiltrated with various types of immunocompetent cells, most of which are adipose tissue macrophages (ATMs) [16,17], the main source of inflammatory factors in adipose tissue. The accumulation of fat is accompanied by an increase in the ATM number and consequently leads to the enhancement of ongoing inflammation via the induction of inflammatory cytokine release by both ATMs as well as by adipocytes [18,19,20,21,22,23].

Although there is no doubt that myokine secretion plays an important role in the pro- and anti-inflammatory balance in the adipose tissue, some aspects of irisin activity still remain unclear. As far as we know, irisin’s impact the inflammatory response of macrophages has not yet been explained. In our previous work we have shown that its action intensifies phagocytosis but at the same time reduces the respiratory burst generated by macrophages [24]. A detailed characteristic of its immunomodulatory role seems to be especially important for the development of therapeutic strategies against low-grade inflammation generated by immunocompetent cells in adipose tissue. Our recent study has demonstrated that irisin significantly downregulates the pro-inflammatory activity of adipocytes (the manuscript under review); therefore, the aim of the present study was to assess the influence of irisin on macrophages. Particular attention has been paid to the activation of the downstream pathway of Toll-like receptor 4 (TLR4) and the release of crucial pro-inflammatory cytokines.

## 2. Results

### 2.1. Irisin Protects Macrophages against Lipopolysaccharide-Induced Injury

**Figure 1:**
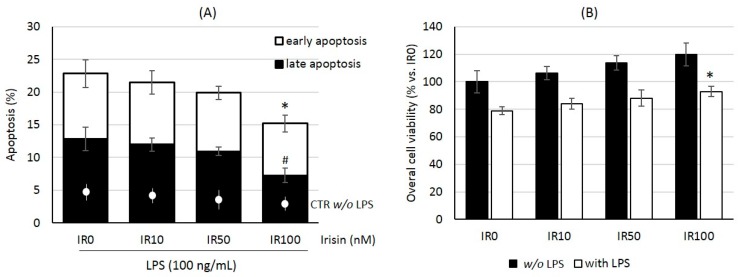
Protective effects of irisin (IR) pretreatment on lipopolysaccharide (LPS)-stimulated macrophage RAW 264.7 cells. The cells were pre-incubated with various doses of irisin (0–100 nM) for 24 h, and after that stimulated with LPS (100 ng/mL) for the next 24 h. Cells viability was tested cytometrically using an Annexin V kit (**A**), where the results are presented as a mean percentage of late apoptotic (solid bars) or early apoptotic cells (open bars) ± S.E. or colorimetrically in the 3-(4,5-dimethyl thiazol-2-yl)-2,5-diphenyl tetrazolium bromide (MTT) test (**B**), where the results are presented as a mean percentage vs. IR0 group. The line within the bars shows the general level of apoptosis measured after 24 h without LPS stimulation. *N* = four to five independent experiments. Statistical significances were determined with the ANOVA with Tukey post hoc analysis. * *p* < 0.05 significance compared with the control group for early apoptosis intensity or overall viability; # *p* < 0.05 significance compared with the control group for late apoptosis intensity.

As the first step of our examination, we tested the effects of irisin preincubation on macrophage viability after LPS stimulation. As shown in Figure 1A, LPS at a dose of 100 ng/mL markedly increased the percentage of apoptotic macrophages, which was evaluated cytometrically with the Annexin V kit, but irisin pretreatment in high concentration (100 nM) significantly reduced these effects (*p* < 0.05). This observation has been confirmed in the MTT test, allowing us to assess the overall cell viability and activity (Figure 1B). Moreover, lower irisin concentrations (10 and 50 nM) were ineffective in both tests. We did not observe any significant influence of irisin pretreatment on the viability of quiescent macrophages, but our results suggest that a high irisin concentration can protect macrophages against LPS-induced injury.

### 2.2. Irisin Has an Impact on TLR4 Expression

**Figure 2:**
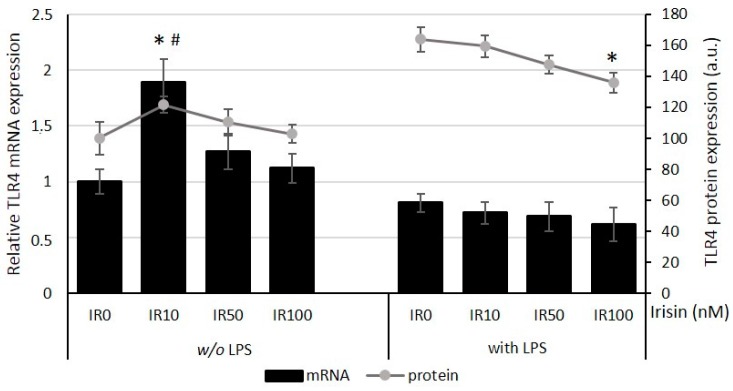
Effects of irisin pretreatment (IR; 0–100 nM) on Toll-like receptor 4 (TLR4) expression on mRNA and protein level. Macrophages RAW 264.7, pretreated with irisin for 24 h, were cultured with and without LPS (100 ng/mL) for an additional 6 h (mRNA expression; rtPCR; bars) or 24 h (protein expression; cytometer; lines). Protein level was expressed as a percentage vs. control group (0 nM) as 100%. Control groups—IR0 with or without (*w*/*o*) LPS. The results are expressed as means + SE of four to five independent experiments (# *p* < 0.05 significance compared with control group for mRNAs expression; * *p* < 0.05 significance compared with control group for protein expression). Statistical significances were determined with the ANOVA with Tukey post hoc analysis.

Taking into account the Toll-like receptor 4 (TLR4) involvement in LPS recognition by macrophages, we evaluated the effects of irisin pretreatment on both TLR4 expression in quiescent as well as in LPS-activated macrophages. As presented in Figure 2 (bars), a 24 h pretreatment of quiescent macrophages with a lower irisin concentration (10 nM) intensified the TLR4 mRNA expression (*p* < 0.05), while higher doses of irisin (50 and 100 nM) were ineffective. These observations were confirmed by cytometric analysis of protein expression quantified after a 24 h preincubation with and without irisin. As presented in Figure 2 (lines), the level of the TLR4 protein was significantly elevated only in the group pretreated with irisin at a low concentration (10 nM; *p* < 0.05).

Moreover, the analysis of TLR4 expressions after LPS stimulation indicated a lack of impact on the mRNA expression after pretreatment with irisin, which was presented in Figure 2 (bars). Nevertheless, a cytometric analysis of the TLR4 protein expression showed a significant reduction in TLR4 protein expression after a high-dose irisin treatment (Figure 2; lines) compared with the control group (0 nM; *p* < 0.05). Pretreatment of macrophages with the lower irisin concentration (10 nM) had no impact on the TLR4 mRNA as well as the protein expression after LPS stimulation. These results showed that a low irisin concentration could potentially improve properties of quiescent macrophages associated with pathogen recognition by the elevation of TLR4 expression. Moreover, our data suggested that a higher irisin concentration, when acting upon the state of macrophage activation, reduced the expression of the TLR4 protein.

### 2.3. Irisin Regulates the Downstream Pathway of TLR4/MyD88

**Figure 3:**
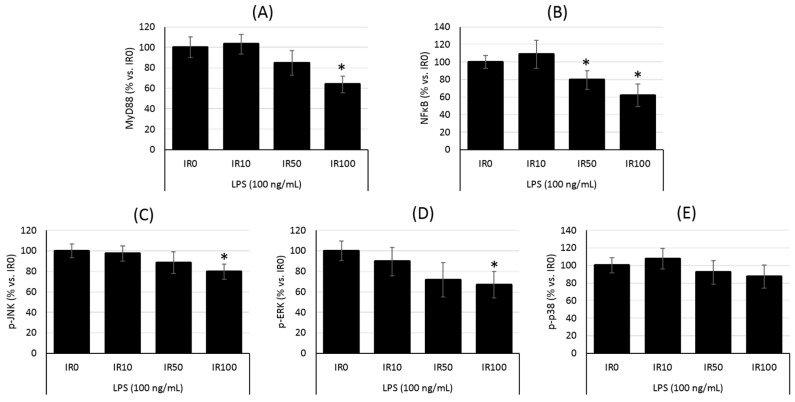
Irisin inhibits the downstream pathway of TLR4/MyD88 in macrophages activated with LPS. RAW 264.7 macrophages were cultured with various irisin concentrations (IR; 0–100 nM) for 24 h and then stimulated with LPS (100 ng/mL). The relative protein levels of MyD88 (**A**); nuclear factor-κB (NF-κB) (**B**), JNK (**C**), ERK (**D**) and p38 mitogen-activated protein kinases (MAPK) (**E**) were evaluated cytometrically and expressed as a percentage vs. control group (irisin 0 nM) presented as a 100%. *N* = four independent experiments. Statistical significances were determined with the ANOVA with Tukey post hoc analysis. * *p* < 0.05 significance compared with control group.

Next, we evaluated the downstream pathway of TLR4 to verify the effects of irisin pretreatment on the signaling cascade activation and pro-inflammatory mediators released by LPS-stimulated macrophages. As presented in Figure 3A, macrophages pretreated with a high irisin concentration (100 nM) manifested significantly less MyD88 expression (*p* < 0.05) compared with control cells (0 nM). Irisin (100 nM) reduced the MyD88 level by nearly 37%, while lower irisin concentrations (10 and 50 nM) induced a slight effect (*p* = 0.05 for 50 nM, and *p* > 0.05 for 10 nM).

Moreover, analysis of the phosphorylation of the crucial components of the TLR4 pathway indicated that the irisin pretreatment resulted in the downregulation of MAPK signaling pathways (Figure 3C–E) and consequently reduced the nuclear factor-κB (NF-κB) activation by 39% (*p* < 0.01; Figure 3B). Particularly, the phosphorylation of JNK and ERK but not p38 kinase was decreased after macrophages were pretreated with a high irisin concentration (100 nM), and the levels were lowered by about 21%, and 34%, respectively (*p* < 0.05). Lower doses of irisin (up to 50 nM) have only a slight impact on kinase phosphorylation (*p* = 0.05 or *p* > 0.05), but after macrophages were pretreated with irisin at 50 nM, a reduction in NF-κB activation was also observed (21%; *p* < 0.05). The lowest irisin concentration (10 nM) had no impact on both the kinases and NF-κB phosphorylation (*p* > 0.05).

### 2.4. Irisin Pretreatment Reduces the Level of Pro-Inflammatory Cytokines

**Figure 4:**
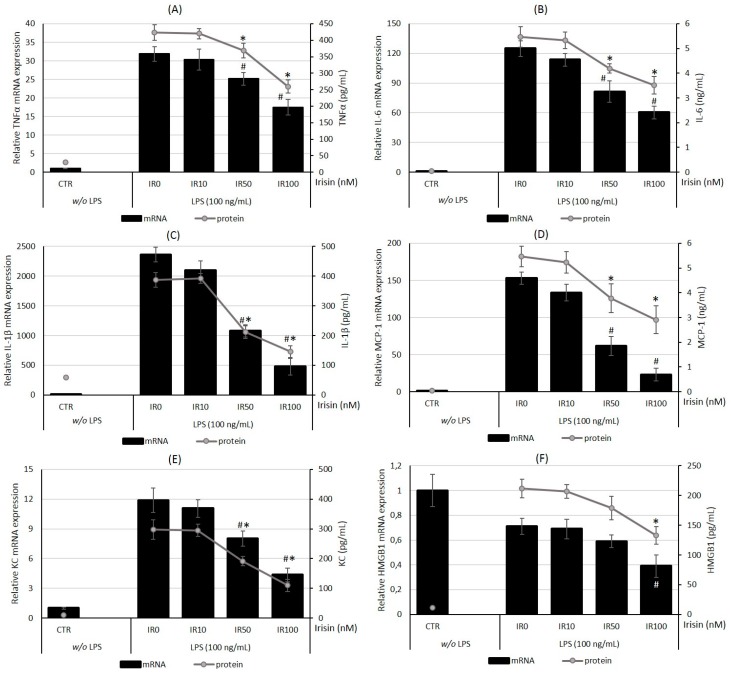
Irisin reduces pro-inflammatory cytokines expression and secretion by LPS-activated macrophages. Macrophage RAW 264.7 cells were pretreated for 24 h with irisin (IR; 0–100 nM) and then stimulated with LPS (100 ng/mL) for 4 h (mRNA expression; rtPCR) or 24 h (cytokine release; ELISA tests). Relative mRNA expression (bars) and protein secretion (lines) of tumor necrosis factor alpha (TNFα) (**A**), interleukin 6 (IL-6) (**B**), interleukin 1β (IL-1β) (**C**), monocyte chemotactic protein 1 (MCP-1) (**D**), keratinocyte chemoattractant (KC) (**E**), and high mobility group box 1 (HMGB1) (**F**) were assessed. CTR—non-stimulated group, quiescent macrophages. The results are expressed as means ± SE of four to five independent experiments (* *p* < 0.05 significance compared with IR0 group for mRNA expression; # *p* < 0.05 significance compared with IR0 group for cytokine release to supernatant). Statistical significances were determined with the ANOVA with Tukey post hoc analysis.

The last step in our investigation was the evaluation of cytokine/chemokine expression and secretion after pretreatment with irisin. Macrophages stimulated with LPS markedly increased both the cytokine mRNA expression as well as the cytokine release measured after 24 h of stimulation (Figure 4). Moreover, a 24 h pretreatment of macrophages with irisin in a dose-dependent manner reduced both the mRNA expression and secretion of TNFα, IL-1β, IL-6, MCP-1, KC and HMGB1. As presented in Figure 4A, higher irisin concentrations (100 and 50 nM) effectively reduced the TNFα mRNA expression by 45% and 22%, and the TNFα release by 40% and 18%, respectively (*p* < 0.01). In the case of IL-6, the mRNA level was lower—about 51% (100 nM) and 35% (50 nM)—while the cytokine content in the supernatant was reduced by 36% and 27%, respectively (*p* < 0.05; Figure 4B). The mRNA level of IL-1β was reduced by 79% by irisin at 100 nM, and by 52% by irisin at 50 nM (*p* < 0.001; Figure 4C). The level of IL-1β released was decreased respectively by 62% and 45% in comparison with the control group (0 nM; *p* < 0.01). Moreover, higher irisin concentrations (100 and 50 nM) effectively reduced MCP-1 mRNA expression by 80%, and 56%, respectively, which was accompanied by the lowering of MCP-1 secretion by 46% and 30%, respectively (Figure 4D).

Furthermore, the analysis of KC distribution in RAW 264.7 macrophages indicated that high irisin concentrations (100 and 50 nM) markedly inhibited the KC mRNA expression by 65% and 33%, respectively (*p* < 0.01), and consequently reduced the level of KC released upon LPS stimulation by 52% (*p* < 0.01) and 28% (*p* < 0.05; Figure 4E).

The effect of irisin on HMGB1 expression was not so prominent (Figure 4F), but irisin at a 100 nM concentration significantly reduced both the mRNA and cytokine levels by 28% and 20%, respectively (*p* < 0.05). In all cases, the lowest irisin concentration (10 nM) did not diminish the level of tested cytokines (*p* > 0.05). Moreover, quantification of mRNA levels in quiescent macrophages indicated that the rest levels of TNFα and IL-1β mRNA expression are downregulated in macrophages pre-incubated with irisin (100 nM) for 24 h, when compared to control cells (0 nM; *p* < 0.05).

## 3. Discussion

Irisin is a peptide that has been widely investigated in the recent years. Numerous studies highlight the beneficial and protective properties of irisin level elevation in obesity, insulin resistance, metabolic disorders [25,26,27] and hepatic steatosis [28]. Moreover, data show that the irisin plasma level may be a predictive factor for such conditions as diabetes mellitus 2 [29], cardiometabolic risk in sedentary lifestyles [30], sarcopenia and carotid arteriosclerosis [31], chronic kidney diseases [32], polycystic ovary syndrome [33] or breast cancer [34]. As reported by Rana et al. [35], irisin level correlated with the patient’s age may predict telomere length. Repeatedly, studies also indicated a correlation between the irisin level and the level of inflammatory factors, suggesting its anti-inflammatory properties [36,37]. Nevertheless, there is still little evidence to explain the direct effect of irisin on the activation of immunocompetent cells [38]. Therefore, research on the mechanisms of action of irisin is particularly needed.

Macrophages, as crucial cells in the first line of our immune defense, play an important role in pathogen elimination and the recruitment of other cells to the place of ongoing inflammation. However, in some pathological conditions, such as, for example, obesity, their excessive activation can lead to the induction of mild inflammation and consequently to associated disease development. In the mechanism of pathogen recognition, a prominent role is assigned to Toll-like receptors (TLRs) which are stimulated by pathogen-associated molecular patterns (PAMPs) as a typical structural motif of bacteria, viruses or fungi [39]. LPS, a component of the outer membrane of Gram-negative bacteria, as a PAMP interacts with the TLR4 receptor and triggers the downstream pathway activation, leading to phosphorylation and translocation of the transcription factor NF-κB, and consequently the secretion of pro-inflammatory mediators [40]. The downstream TLR4 pathway, through interaction of several protein complexes, leads to the activation of the MyD88-dependent or MyD88-independent pathways and consequently to pro-inflammatory cytokines or interferon type I being released, respectively [39]. At this point we should pay attention to two aspects analyzed in this work; firstly on irisin’s influence on resting macrophages, and secondly on macrophages stimulated by LPS. Analysis performed on quiescent cells indicated that the pretreatment of macrophages with a low irisin concentration results in the enhancement of TLR4 expression, which may suggest the increased ability of macrophages to recognize potential pathogens. However, it should be taken into account that TLR4 is a highly glycosylated protein which affects its functionality in terms of LPS recognition [41]. Our next studies suggest that irisin’s influence on glycosylation and the formation of TLR4 glycoforms are possible (study under development). However, further studies are needed to explain and verify these aspects. Moreover, diminished release of TNFα and IL-1β in the resting stage should also be emphasized. Other analyzed factors were not significantly different, but the reduction of TNFα and IL-1β release draws attention to a potential reduction in the spontaneous pro-inflammatory activity of these cells. Our previous study [25] indicated, moreover, that irisin enhances macrophage proliferation and has a positive impact on the phagocytosis of bacteria while also inhibiting respiratory burst generation. In the state of macrophage activation by LPS stimulation, irisin pretreatment induced prominent anti-inflammatory effects, closely connected with the TLR4/MyD88 downstream pathways. We observed a dose-dependent reduction in TLR4 protein expression after irisin pretreatment, which was accompanied by a reduction in the MyD88 level. It should be noted that the TLR4/MyD88 pathway activates signaling such as the mitogen-activated protein kinase (MAPK) pathway as well as the NF-κB pathway. This study indicates that irisin modulates both the activation of NF-κB and the MAPK pathway, leading to a reduction in the phosphorylation of the mentioned factors. The anti-inflammatory effect of the irisin pretreatment, suggested in this paper, was manifested mainly by the lowering of the expression and secretion of key inflammatory cytokines. We observed a reduction in TNFα, IL-1β, IL-6, MCP-1, KC and HMGB1 levels, which can be explained by the above-mentioned inhibition of the TLR4/MyD88 pathway. Irisin’s impact on the inhibition of TNFα, IL-1β, and IL-6 was previously mentioned by Dong et al. [38], who also noted the promotion of the alternative polarization of macrophages undergoing irisin stimulation. Moreover, our study demonstrated that irisin inhibits the expression and release of HMGB1, a nuclear DNA-binding protein which can be actively secreted by stimulated macrophages, or passively by damaged or necrotic cells [42]. As a late mediator of inflammation, HMGB1 induces an LPS-independent activation of NF-κB [43] and consequently enhances the secretion of pro-inflammatory factors, including TNFα. Because of the HMGB1 action, amplification of the inflammatory response could be observed [44]. In our study, the level of HMGB1 was effectively reduced by a high irisin concentration, but the mechanisms of this inhibition should be examined in the future.

In conclusion, as presented in our study, irisin alleviates the inflammatory activation of macrophages stimulated by LPS. The anti-inflammatory effect of irisin, observed in this study, is mediated by inhibition of the downstream pathway of TLR4/MyD88, which is connected with the suppressed phosphorylation of MAPK and consequently a lower NF-κB activation. As a result of these changes, we observed the reduction in both the expression and release of pro-inflammatory cytokines, such as IL-1β, TNFα, IL-6, KC, MCP-1 and HMGB1. Our results may suggest that beneficial and anti-inflammatory properties of physical activity, as well as the potential protective effects of irisin against the development of diseases associated with obesity, may at least in part be associated with irisin’s anti-inflammatory properties.

## 4. Materials and Methods

### 4.1. Chemicals and Materials

DMEM medium, antibiotics (streptomycin and penicillin), fetal bovine serum (FBS) were purchased from PAA (Pasching, Austria). Lipopolysaccharide (LPS) was purchased from Sigma-Aldrich (St. Louis, MO, USA). Irisin was purchased from Cayman. Annexin V kit, Fc block antibodies, Cytofix/Cytoperm solution, PerCP-Cy5.5 streptavidin, Cytometric Bead Arrays for JNK, ERK and p38 quantification were acquired from BD Biosciences Pharmingen (San Diego, CA, USA). Phospho-NF-κB (ser536) Alexa Fluor 647 Conjugate rabbit monoclonal antibody was acquired from Cell Signaling Technology (Beverly, MA, USA). Elisa kit for HMGB1 and IL-1β were purchased from IBL International (Hamburg, Germany). RNeasy Plus Mini Kit (74134) for RNA isolation was obtained from Qiagen (Hilden, Germany). High Capacity RNA-to-cDNA Kit (4387406) for reverse transcription and TaqMan Gene Expresson Master Mix (4369026) for real time PCR were obtained from Applied Biosystems (ThermoFisher; Foster City, CA, USA).

### 4.2. Cell Culture and Experimental Design

The study was conducted on a murine macrophage-like RAW 264.7 cell line (European Type Culture Collection; ETCC, Sigma), free of mycoplasma contamination. The cells were cultured under standard conditions (37 °C, 5% CO_2_) in a DMEM medium supplemented with 1% of antibiotic, 10% of fetal bovine serum, and irisin (0–100 nM) for 24 h, and after that the cells were stimulated with LPS (100 ng/mL; *Escherichia coli*, serotype 0111: B4). Fresh cells were used for MTT test, cytometric assessment and RNA isolation. Supernatants were frozen (−60 °C) for future quantification of cytokine levels.

### 4.3. Colorimetric Examination of Overall Cell Viability

The total cell viability and activity was measured by assaying the reduction of 3-(4,5-dimethyl thiazol-2-yl)-2,5-diphenyl tetrazolium bromide (MTT) to formazan, according to the manufacturer’s instruction and quantify on a spectrophotometer (Expert Plus, ASYS/Hitech, Eugendorf, Austria).

### 4.4. Quantitative Real-Time PCR Assay

Quantitative Real time PCR was used to assess TLR4, TNFα, IL-1β, IL-6, MCP-1, KC and HMGB1 gene expression. RNA was extracted from RAW 264.7 cells using RNeasy Plus Mini Kit (Qiagen, 74134) and reverse transcription was carried out using the High Capacity RNA-to-cDNA Kit (Applied Biosystems, 4387406) according to the manufacturer’s protocols. Real time PCR was performed using TaqMan Gene Expresson Master Mix (Applied Biosystems, 4369026) in Step One Plus thermocycler (Applied Biosystems). The expression of analyzed genes was normalized to the expression level of glyceraldehyde-3-phosphate dehydrogenase (GAPDH) used as a housekeeping gene. mRNA expression of each sample was determined in four-five (separate RNA isolation) using the 2^−ΔΔ*C*t^ method.

### 4.5. Flow Cytometric Analysis

The irisin impact on macrophage apoptosis was determined using an Annexin V kit according to the protocol provided by the producer. For the analysis, 10,000 cells were acquired. Cells were previously pretreated with irisin (0–100 nM) for 24 h and subsequently with LPS (100 ng/mL) for additional 24 h.

The level of MyD88 and NF-κB were detected by flow cytometry after labeling with a fluorescent antibody. Briefly, after a 24 h preincubation with irisin (0–100 nM), cells were stimulated with LPS for additional 24 h. After that, cells were detached, blocked with Fc-block (0.5 mg/mL; 1:200; 20 min; 4 °C), fixed, and permeabilized according to the manufacturer’s instructions (Cytofix/Cytoperm) and treated with an appropriate antibody. For MyD88 detection, the biotin anti-MyD88 antibody (0.2 mg/mL; 1:200; 20 min; 4 °C) and PerCP-Cy5.5 streptavidin (0.2 mg/mL; 1:200; 20 min; 4 °C) were used; for the detection of the phosphorylated form of NF-κB, cells were stained with phosphor-NF-κB (Ser536) Alexa Fluor 647 Conjugate rabbit monoclonal antibody (0.1 mg/mL; 1:100; 20 min). All antigens were detected separately. For data analysis, 10,000 pre-labeled cells were collected.

The levels of the phosphorylated forms of kinases JNK, ERK and p38 were determined using a commercial Cytometric Bead Arrays according to the manufacturer’s protocol.

All the samples were acquired and analyzed on a FACScan flow cytometer (FACSCaliburTM; BD Biosciences, San Diego, CA, USA) using CBA and CellQuest software (BD Biosciences, San Diego, CA, USA).

### 4.6. Elisa Examination of Cytokine/Chemokine Release

The levels of such cytokine as TNFα, IL-1β, IL-6, MCP-1, KC and HMGB1 were quantified using a commercial ELISA Kit, according to the manufacturer’s instruction and measured on a spectrophotometer (Expert Plus, ASYS/Hitech, Eugendorf, Austria). All cytokines were measured separately in supernatants collected after 24 h of LPS stimulation.

### 4.7. Statistical Analysis

Data were tested for normality of the distribution, and differences among groups were determined using ANOVA with Tukey post hoc analysis. All data were expressed as means ± standard error (X ± SE) with the level of statistical significance (*p*) set at 0.05.

## Abbreviations

ATMs	Adipose tissue macrophages
FNDC5	Fibronectin type III domain containing 5 protein
HMGB1	High mobility group box 1
IL-1β	Interleukin 1β
IL-6	Interleukin 6
KC	Keratinocyte chemoattractant
LPS	Lipopolysaccharide
MAPKs	Mitogen-activated protein kinases
MCP-1	Monocyte chemotactic protein 1
MyD88	Myeloid differentiation primary response protein
NF-κB	Nuclear factor-κB
p-ERK	Phospho extracellular signal–regulated kinase
p-JNK	Phospho c-Jun N-terminal kinase
TLR4	Toll-like receptor 4
TNFα	Tumor necrosis factor α
WAT	White adipose tissue
